# Thioredoxin-1 Regulates Microglia Polarization to Ameliorate Neuroinflammation and Cognitive Impairment Following Tumor-Therapeutic Ovariectomy

**DOI:** 10.2174/0118715303426987251204044743

**Published:** 2026-01-16

**Authors:** Xiang Zhang, Congxia Pan, Qianyun Xu, Jing Wang, Chaojun Wang

**Affiliations:** 1 Department of Anesthesiology and Oncology, Shanghai Medical College, Fudan University Shanghai Cancer Center, Shanghai 200032 , China;; 2 Department of Reproductive Medicine, Ren Ji Hospital, Shanghai Jiao Tong University School of Medicine, Shanghai 200135, China;; 3 Shanghai Key Laboratory for Assisted Reproduction and Reproductive Genetics, Shanghai 200135, China

**Keywords:** Trx-1, microglial activation, neuroinflammation, menopause, cognitive impairment, tumor-therapeutic ovariectomy

## Abstract

**Introduction:**

Neuroinflammation is a key driver of cognitive deficits after surgical menopause, a state commonly triggered by oophorectomy for oncological purposes. This intervention is routinely performed in premenopausal patients with hormone receptor-positive (HR+) breast cancer, constitutes foundational therapy for epithelial ovarian carcinoma, and is indicated for risk reduction in carriers of hereditary mutations such as breast cancer susceptibility gene 1/2 (BRCA1/2). As highly efficient immune cells, microglia play a central role in the onset of neurodegenerative diseases. Our study explored the role of thioredoxin-1 (Trx-1) in suppressing microglial activation and its potential for ameliorating postmenopausal cognitive decline.

**Methods:**

Female C57BL/6J mice underwent ovariectomy (OVX) to model tumor-therapeutic oophorectomy. They were intraperitoneally injected with recombinant human Trx-1 (rhTrx-1) at a dose of 200 μg/30 g or PBS once weekly for five weeks following OVX. A Y-maze active avoidance task and trace fear conditioning were used to assess cognitive function. Levels of neuroinflammation were evaluated through immunofluorescence, enzyme-linked immunosorbent assay (ELISA), and real-time polymerase chain reaction (PCR). Trx-1 levels in the hippocampus of OVX mice were measured by western blot and immunohistochemical analysis. Furthermore, the impact of Trx-1 on microglial activation was assessed *in vitro*.

**Results:**

OVX induced the production of inflammatory factors and microglial activation in the brain, resulting in cognitive deficits in mice. However, intraperitoneal injection of rhTrx-1 inhibited these effects by improving cognitive function, reducing inflammatory cytokines, and promoting microglial polarization. *In vitro* studies also showed that rhTrx-1 attenuated lipopolysaccharide (LPS)-stimulated microglial activation through mitogen-activated protein kinase (MAPK) pathway signaling.

**Discussion and Conclusion:**

Due to its inhibitory effect on neuroinflammation and microglial activation, Trx-1 may represent a promising therapeutic candidate for managing cancer therapy-related cognitive impairment, particularly in patients undergoing estrogen-deprivation therapies such as oophorectomy.

## 1 INTRODUCTION

Oophorectomy, the surgical removal of the ovaries, is a cornerstone intervention in oncology, deliberately inducing menopause to treat and prevent hormone-sensitive malignancies. This procedure is fundamentally characterized by two oncological rationales: therapeutic castration for active disease and prophylactic menopause for cancer risk reduction. Its most pivotal role is in the management of premenopausal hormone receptor-positive (HR+) breast cancer, where oophorectomy is employed as a definitive ablative therapy. By abruptly triggering a postmenopausal state through the elimination of ovarian estrogen, it effectively suppresses tumor growth and improves survival outcomes [1]. Additionally, the procedure forms the foundation of surgical care for epithelial ovarian cancer, where it is integral to comprehensive staging and cytoreductive operations [2].

In the realm of cancer prevention, oophorectomy is the most effective measure to mitigate risk in individuals with hereditary cancer syndromes. For healthy women carrying high-penetrance genetic mutations such as breast cancer susceptibility gene 1 (BRCA1) and BRCA2, elective risk-reducing salpingo-oophorectomy is a standard recommendation. This intervention, which purposefully initiates premature menopause upon completion of childbearing, dramatically reduces the future incidence of both ovarian and breast cancers, thereby lowering cancer-specific mortality [3].

As survival rates improve, the oncological paradigm is shifting from a singular focus on survival to the comprehensive management of treatment sequelae [4]. An accumulating body of evidence demonstrates that women who undergo surgical menopause at an early age are significantly more susceptible to dementia and cognitive dysfunction [5-7]. Dementia and cognitive decline have emerged as significant public health concerns, with epidemiological investigations indicating that the prevalence of dementia is projected to triple by 2050. Evidence suggests that midlife may represent a crucial stage in the natural progression of dementia, particularly among women [8]. Understanding how menopause affects cognitive function during midlife and exploring effective therapeutic interventions remain crucial. Estrogen participates in numerous cellular pathways within the brain, sustaining normal brain activity [9]. Despite its neuroprotective effects, certain clinical trials have revealed that menopausal hormone therapy may not be beneficial or could even be detrimental to cognitive function in postmenopausal women [10-12]. Therefore, identifying effective treatments to prevent and manage menopausal cognitive impairment is imperative.

Inflammation has been defined as a biomarker of accelerated aging, indicating that the presence of chronic low-grade inflammation contributes to age-related neurodegeneration [13, 14]. Older organisms are prone to experiencing persistent low-grade inflammatory conditions in their cells and tissues [15]. This phenomenon, referred to as immune aging, has been linked to the development of neurodegenerative disorders [16]. Neuroinflammation is a prevalent feature in most neurodegenerative disorders and has been identified as an underlying cause of cognitive impairment [17]. Microglia can initiate and propagate neuroinflammation, contributing to neurotoxic effects through a feedforward inflammatory loop that ultimately leads to neurodegeneration [18, 19]. Inhibition of microglial activation and the resulting excessive inflammatory response may potentially alleviate the severity of cognitive impairment following menopause.

As a redox-active protein, thioredoxin (Trx) is widely distributed throughout the body and serves as a protective protein under stress. Trx-1, a highly conserved 12-kDa protein, exhibits antioxidant effects [20]. Zhang *et al*. found that Trx-1 could improve learning and memory dysfunction in a mouse Parkinson’s disease model [21]. Nevertheless, it remains unclear whether Trx-1 affects inflammation and cognitive impairment following menopause. To recapitulate the hormonal ablation achieved by cancer therapeutic oophorectomy in patients, we utilized the rodent model of ovariectomy (OVX), its standard preclinical counterpart [22]. Therefore, we investigated whether administration of Trx-1 could suppress microglial activation and ameliorate cognitive decline in OVX mice.

## 2 MATERIALS AND METHODS

### 2.1 Animals

Laboratory animals were cared for and used in accordance with the rules established by the Institutional Animal Care and Use Committee of Fudan University Shanghai Cancer Center (approval number: FUSCC-IACUC-S2023-0048). Twelve-week-old female C57BL/6J mice (n=60, 20 g) were purchased from Shanghai JieSiJie Laboratory Animals Co., LTD (Shanghai, China). The sample size was determined based on previous studies [23, 24]. Five mice were housed per cage (humidity: 40%, temperature: 22.0°C) with ad libitum access to food and water. The study design is briefly illustrated in Fig. (**1**). Animal treatments and analyses were performed blinded to group assignment. All procedures were prepared prior to the study. The order of measurements and cage locations were randomized. All mice were euthanized by carbon dioxide (CO_2_) asphyxiation, and only mice that were healthy were included in the study.

**Fig. (1):**
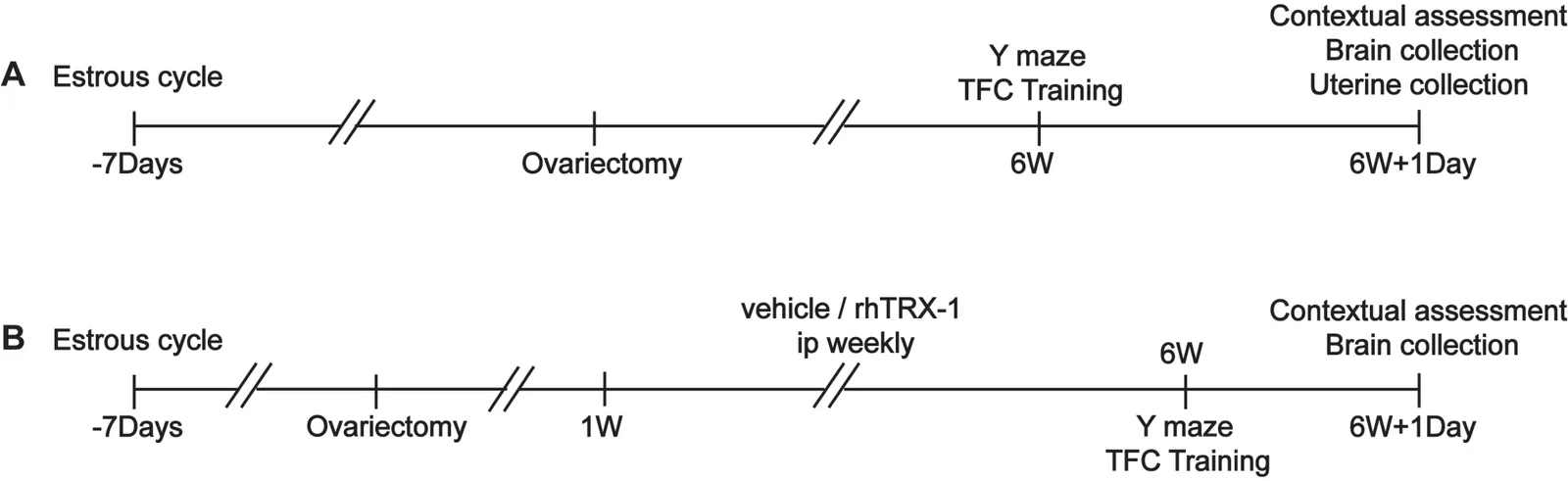
Study design *in vivo*. (**A**) Experiment 1: All mice were included in the following experiment according to the estrous cycle. Seven days later, the OVX surgery was performed. All mice received a contextual assessment 6 weeks after surgery. Tissues were then obtained. (**B**) Experiment 2: Same as experiment 1, mice were intraperitoneally injected with rhTrx-1 or PBS following OVX.

Mice with regular estrous cycles underwent bilateral ovariectomy (OVX, n=10) or a sham procedure (n=10) under isoflurane anesthesia using a random number table for group assignment (Fig. **1A**). Cognitive function was assessed six weeks later using the Y-maze test and trace fear conditioning. Brain and uterus samples were then collected.

Mice were randomly assigned to four groups: (1) Sham group (n=10); (2) rhTrx-1-treated Sham mice group (rhTrx-1 group, n=10); (3) OVX mice treated with vehicle only (OVX group, n=10); and (4) OVX mice treated with rhTrx-1 (OVX+rhTrx-1 group, n=10, Fig. **1B**). Briefly, a total of forty mice underwent bilateral OVX (n=20) or a sham procedure (n=20). One week later, mice received intraperitoneal injections of either 200 μg/30 g rhTrx-1 or phosphate-buffered saline (PBS) once weekly for five weeks. After behavioral experiments, mice were sacrificed, and brains were collected. Hippocampal tissue was rapidly frozen in liquid nitrogen and stored at -80 °C for subsequent analyses.

### 2.2 Postmenopausal Model

The OVX mouse is an established rodent model that replicates the pathophysiology of surgical menopause, a state directly induced by endocrine therapy for hormone-sensitive malignancies like breast cancer [22]. Following anesthesia with isoflurane (2%-4% with 100% oxygen), the ovaries of the OVX group were exposed and excised through a dorsal incision, while those of the sham group were exposed and replaced without removal.

### 2.3 Experimental Groups and rhTrx-1 Administration

In the *in vivo* study, female mice were assigned to one of the following groups at random (n=10): (1) Sham group; (2) rhTrx-1-treated Sham mice (rhTrx-1 group); (3) OVX mice treated with vehicle only (OVX group); and (4) OVX mice administered with rhTrx-1 (OVX+rhTrx-1 group). The OVX mice received intraperitoneal injections of either 200 μg/30 g rhTrx-1 from MCE, China, or PBS once a week for five weeks. The dosages were selected on the basis of an earlier study [25]. In the *in vitro* study, BV-2 microglial cells were treated with 100 ng/ml lipopolysaccharide (LPS) or PBS (Control). Cells were exposed to 25 μg/ml rhTrx-1 for a duration of 30 minutes before the administration of LPS (LPS+rhTrx1) or PBS (rhTrx1). The selection of this dosage was made on the basis of the results in a previous investigation conducted by Tian *et al,* [26].

### 2.4 Behavioral Tests

The experimenter was blinded to the group assignments throughout the behavioral testing and analysis phases. All training procedures were performed consistently, with sessions conducted at the same time of day in a controlled environment to minimize variability. Any mice that did not meet the pre-established training criteria were excluded from the data analysis.

### 2.5 Trace Fear Conditioning (TFC)

Hippocampal-dependent memory in rodents was assessed using the trace fear conditioning (TFC) method according to a previous study [27]. Mice underwent a conditioning protocol in which they were trained to associate a tone (conditioned stimulus) with a foot shock (unconditioned stimulus). Mice initially explored the conditioning chamber for 100 seconds to familiarize themselves with the context. An auditory cue consisting of a 5 kHz tone at 80 dB was then presented for 20 seconds, followed immediately by a 0.8 mA foot shock lasting 2 seconds. After a 100-second interval, the procedure was repeated, and mice were removed from the chamber 30 seconds later. Contextual memory was assessed in the absence of any cues (tone or shock). Freezing time was recorded for 300 seconds using video recording and analyzed with specialized software. Memory deficits were indicated by reduced freezing time.

### 2.6 Y-Maze Active Avoidance Task

This study employed a modified active avoidance paradigm based on the Y-maze structure (Y-maze active avoidance task) to assess associative learning and spatial avoidance abilities in mice, as described in our previous study [27]. The maze consisted of three arms (I–III, each 30 × 5 × 20 cm) converging on a central equilateral triangular region (region 0). The distal end of each arm was equipped with a lamp. Illumination indicated the safe region, while electrical foot stimulation (25 V) was applied in the other regions.

Mice were initially placed at the end of a randomly selected arm and allowed to explore the maze for three minutes without any external stimuli to acclimate. The test was then initiated, with the lighted arm (designated as the safe area) serving as the new starting point. The direction of both the safe and stimulation areas was altered randomly during the task. A trial was considered successful when a mouse reached the designated safe area within 10 seconds. Mouse responses were observed until the animal reached the lighted arm before the next stimulation was applied. Learning was considered achieved when a mouse correctly responded nine times out of ten trials. The total number of stimuli required to reach this criterion during training was recorded to evaluate learning ability.

### 2.7 Immunohistochemical Analysis

Mice were humanely euthanized, and their cerebral tissues were harvested. The tissues were subsequently fixed and subjected to histopathological examination *via* immunohistochemistry [27, 28]. Immunohistochemical and immunofluorescent analyses were then performed on brain sections using polyclonal antibodies, including ionized calcium-binding adapter molecule 1 (Iba1, 1:100, Wako, Japan) and Trx-1 (1:100, Proteintech, China).

### 2.8 BV-2 Culture

BV-2 immortalized murine microglia cells (CVCL_0182) were obtained from Haixing Biosciences (Suzhou, Jiangsu, China). The catalogue number was TCM-C718. Iba1 was used to determine the cell purity. BV-2 were cultured with fetal bovine serum (FBS), penicillin, and streptomycin in Dulbecco's Modified Eagle's medium (DMEM) (Gibco, USA).

### 2.9 Immunofluorescence

We fixed BV2 microglia with 4% paraformaldehyde, 0.1% Triton X-100, and then 5% bovine albumin. After incubating with Iba1 and Trx-1 antibodies (1:200) for a night, cells were treated with secondary antibodies (1:200) for 1 hour. Nuclei staining was performed using 4',6-diamidino-2-phenylindole (DAPI) (Servicebio, China).

### 2.10 Real-Time PCR

A Foregene RNA Extraction Kit (China) was used to acquire total RNA from hippocampus under microdissection and BV-2 microglial cells. RNA concentration and purity were determined by the A260/A280 absorbance ratio. The cDNA was synthesized by RT SuperMix Perfect for quantitative polymerase chain reaction (Vazyme, China). The reverse transcription procedure was performed in a 20 μl reaction system. The protocol consisted of 15 minutes at 37°C, followed by 5 seconds at 85°C, with final storage at 4°C. Negative control reactions (omitting reverse transcriptase) were included to evaluate potential genomic DNA contamination in the RNA samples. Reverse-transcription quantitative real-time polymerase chain reaction (RT-qPCR) was employed to measure target gene expression levels using Taq Pro Universal SYBR qPCR Master Mix (Vazyme, China). Amplification reactions (20 μL total volume) utilized the following protocol: 95°C for 30 s, followed by 40 cycles at 95°C for 5 s and 60°C for 30s.

Primers were sequenced below: mouse cluster of differentiation 86 (CD86) 5′- GACCGTTGTGTGTGTTCTGG-3′ and 5′ -GATGAGCAGCATCCAAGGA-3′; mouse cluster of differentiation 206 (CD206) 5′-CAAGGAAGGTTGGCATTTGT-3′ and 5′-CCTTTCAGTCCTTTGCAAGC-3′; mouse suppressor of cytokine signaling 3 (SOCS3) 5′-TGCAGGAGAGCGGATTCTACC-3′ and 5′-TGACGCTCAACGTGAAGAAG-3′; mouse GAPDH 5′ -AGGCCGGTGCTGAGTATGTC-3′ and 5′-TGCCTGCTTCACCACCTT CT-3′. All primers were produced and purified by reversed-phase chromatography through GenScript. The mRNA expression levels were quantitatively analyzed using the 2−ΔΔCT method, with GAPDH employed as the endogenous control for data normalization. Each qPCR experiment was analyzed in triplicate.

### 2.11 Western Blot Analysis

Total protein was collected from hippocampal tissues and microglia. Using a BCA kit to determine protein concentration, 40 μg/lane was kept apart by SDS-PAGE gels and transferred to polyvinylidene difluoride membranes. Following 1 hour of incubation with 5% bovine serum albumin (BSA), membranes were treated with several antibodies, including Trx-1 (1:5000), c-Jun N-terminal kinase (JNK, 1:5000), phospho-JNK (1:2000), GAPDH (1:20000) from Proteintech, and p38 (1:1000), phospho-p38 (1:1000) from Cell Signaling Technology.

### 2.12 Tumor Necrosis Factor α (TNF-α) and Interleukin- 1β (IL-1β) Measurement

The levels of TNF-α and IL-1β from rodent hippocampus tissue were quantified by a commercially available Enzyme-Linked Immunosorbent Assay (ELISA) kit.

### 2.13 Statistical Analysis

Results from three independent experiments represented mean ± standard deviation (SD) and were analyzed using SPSS 19.0. Differences between the two groups were analyzed by either independent-sample Student's t-tests or Mann-Whitney U tests. A one-way ANOVA and then Turkey's post hoc test were employed to compare differences among groups. Statistical significance was represented as *P* < 0.05.

## 3 RESULTS

### 3.1 OVX Induced Cognitive Impairment in Mice

**Fig. (2):**
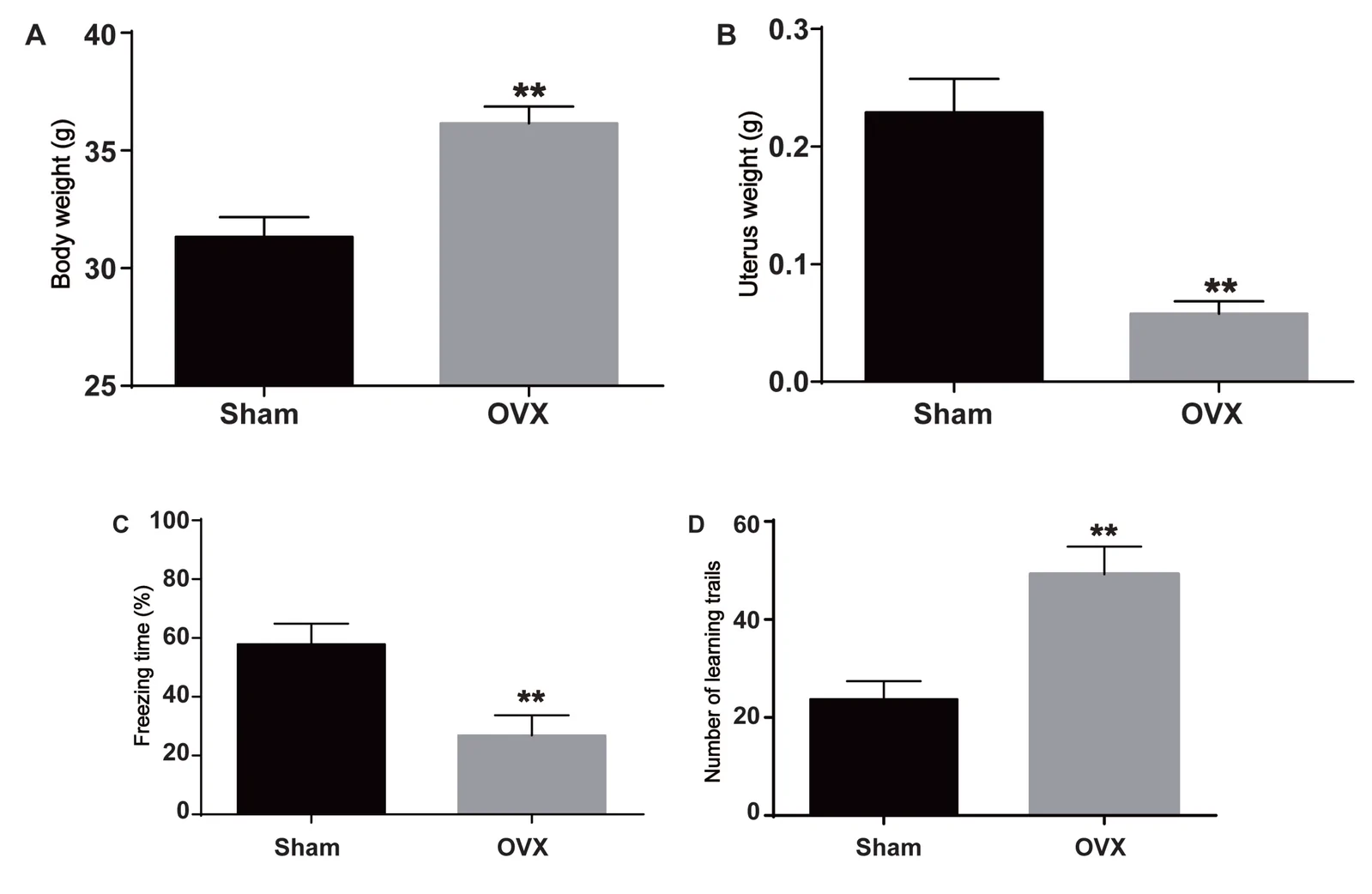
The influence of OVX on cognitive function, body, and uterine weight. (**A**) Body weight was tested. (**B**) Uterine wet weight was measured. (**C**) Freezing time was measured to assess the contextual fear response. (**D**) The Y-maze test was conducted. Error bars: ± SD; Statistical test: Student's t-test (A, C, D) or Mann-Whitney U test (B), **P<0.01 *versus* Sham mice; Sample size: n = 10 mice per group.

The OVX mice had increased body weight (Fig. **2A**) and lower uterine wet weight compared to the sham mice (Fig. **2B**), but the two groups had similar body weight.

To study the effect of OVX on cognition in mice, a contextual assessment and a Y-maze active avoidance task were carried out 6 weeks post-surgery. As depicted in Figs. (**2C**, **D**), a significant reduction in the freezing time and an increase in the number of learning trials were observed following OVX. These findings suggest that menopause may lead to cognitive dysfunction.

### 3.2 OVX-induced Neuroinflammation and Microglial Activation in the Hippocampus

**Fig. (3):**
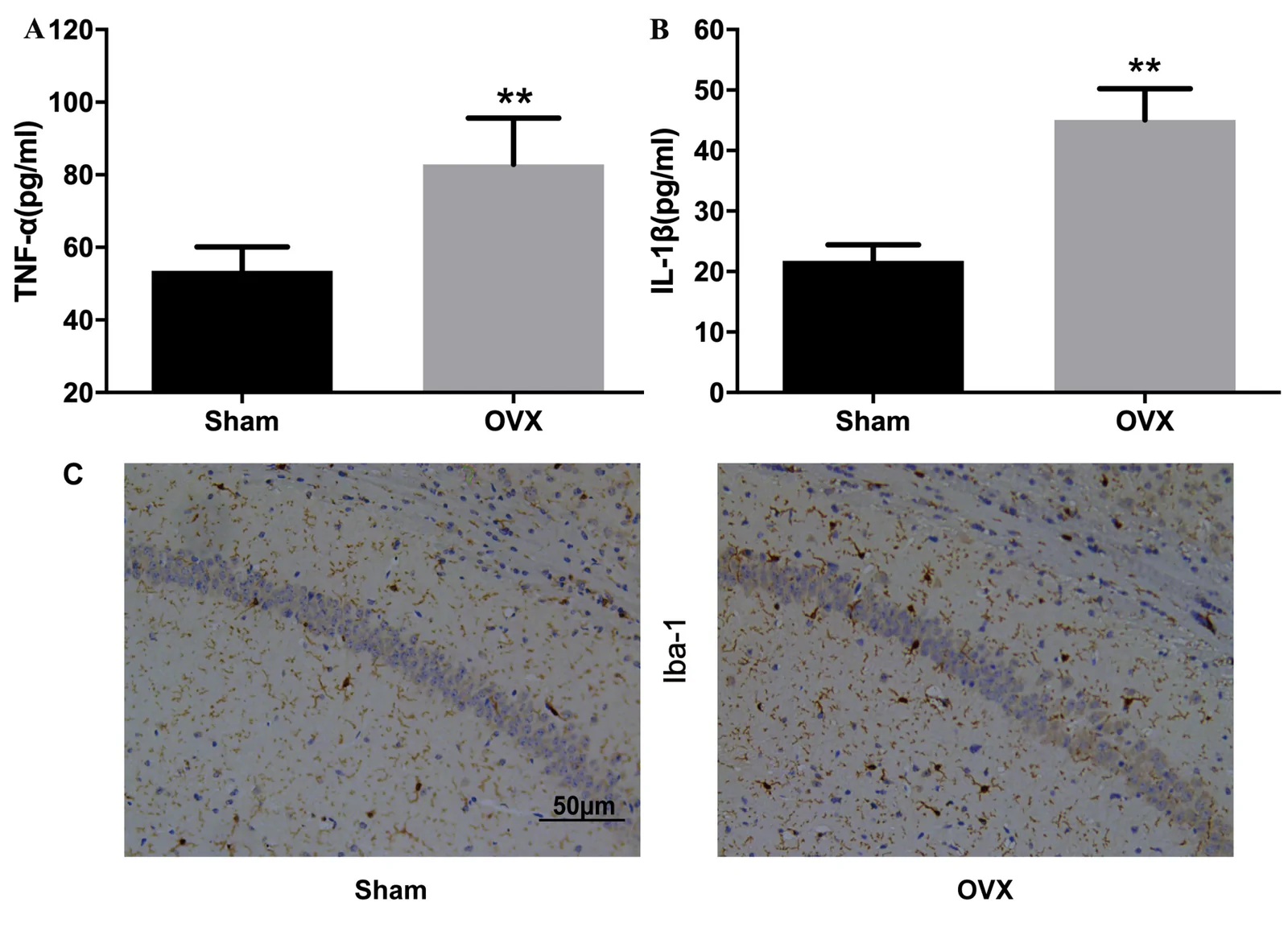
OVX induced pro-inflammatory cytokine release and microglial activation. (**A, B**) The levels of TNF-α and IL-1β, two kinds of pro-inflammatory factors, were measured by ELISA. Error bars: ± SD; Statistical test: Student's t-test, **P<0.01 *versus* Sham mice; Sample size: n=9 mice for Sham group and n=10 mice for OVX group. (**C**) Iba-1immunostaining in the CA1 area of the hippocampus was used to assess the activated microglia. Scale bar = 50 μm.

Given that neuroinflammation has been recognized as one of the key pathological mechanisms in neurodegenerative diseases, we assessed the levels of proinflammatory cytokine secretion and microglial activation in the mouse hippocampus. OVX mice had significantly elevated TNF-α and IL-1β levels in comparison to the sham group (Figs. **3A**, **B**). Meanwhile, the OVX mice showed significant microglial activation in their Cornu Ammonis 1 (CA1) area (Fig. **3C**), indicating that OVX can induce neuroinflammation and microglial activation.

### 3.3 OVX Downregulated the Expression of Trx-1 in the Hippocampus

**Fig. (4):**
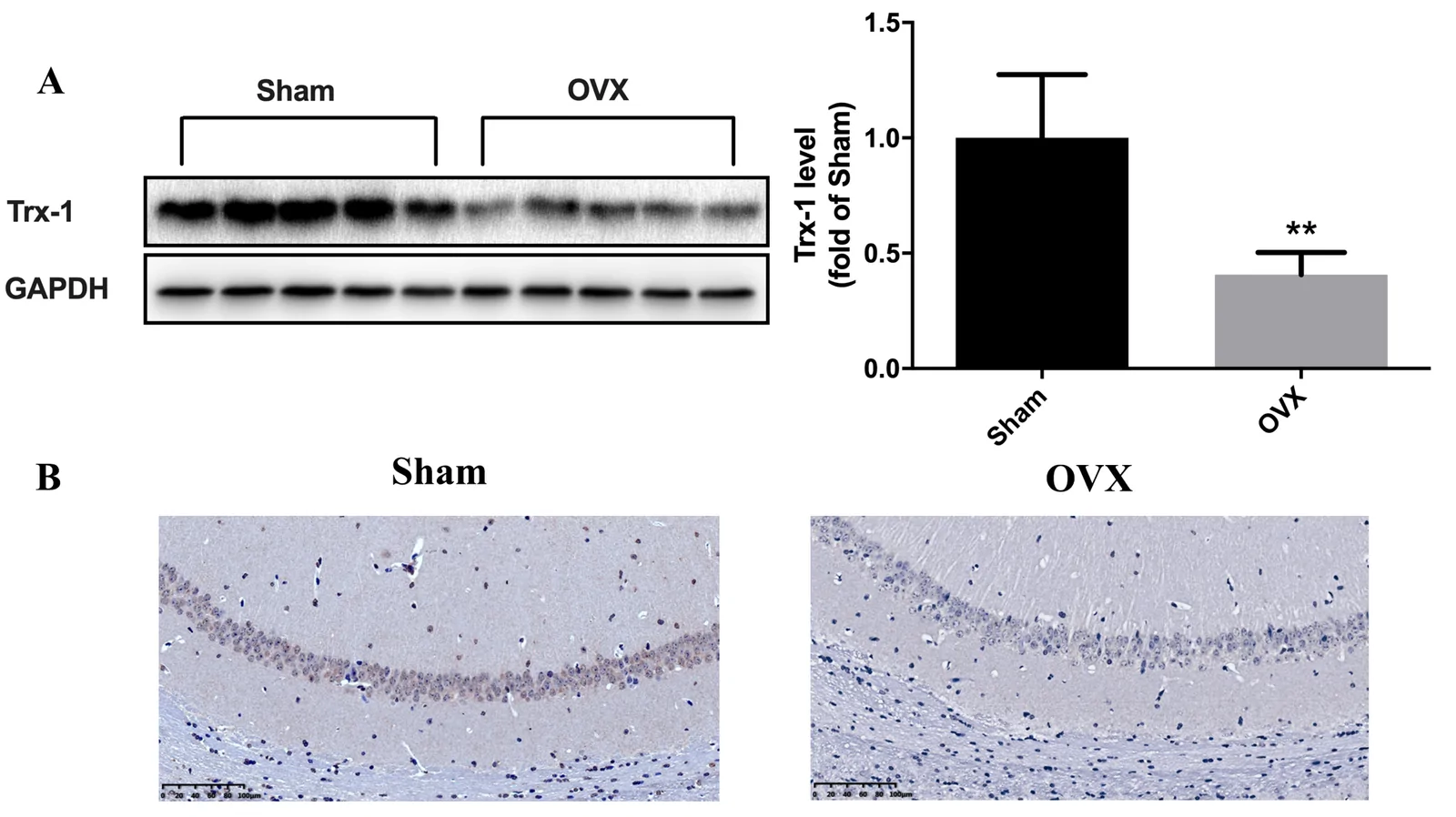
Downregulation of Trx-1 in the hippocampus of OVX mice. (**A**) Trx-1 level in the hippocampus was analyzed by Western blotting. Error bars: ± SD; Statistical test: Student's t-test, **P<0.01 *versus* Sham mice; Sample size: n=5 mice per group. (**B**) Immunohistochemistry was used to detect the level of Trx-1 in the hippocampus in mice. Scale bar = 100 μm.

To investigate the potential involvement of Trx-1 in OVX-induced neuroinflammation, we assessed the expression level of Trx-1 in the hippocampus. OVX led to a significant reduction in the Trx-1 levels in the hippocampus (Figs. **4A**, **B**). These findings suggest that the reduction of Trx-1 may be associated with OVX-induced neuroinflammation, highlighting its therapeutic role of cognitive impairment related to menopause.

### 3.4 Administration of rhTrx-1 Ameliorated Neuroinflammation and Cognitive Impairment Induced by OVX

**Fig. (5):**
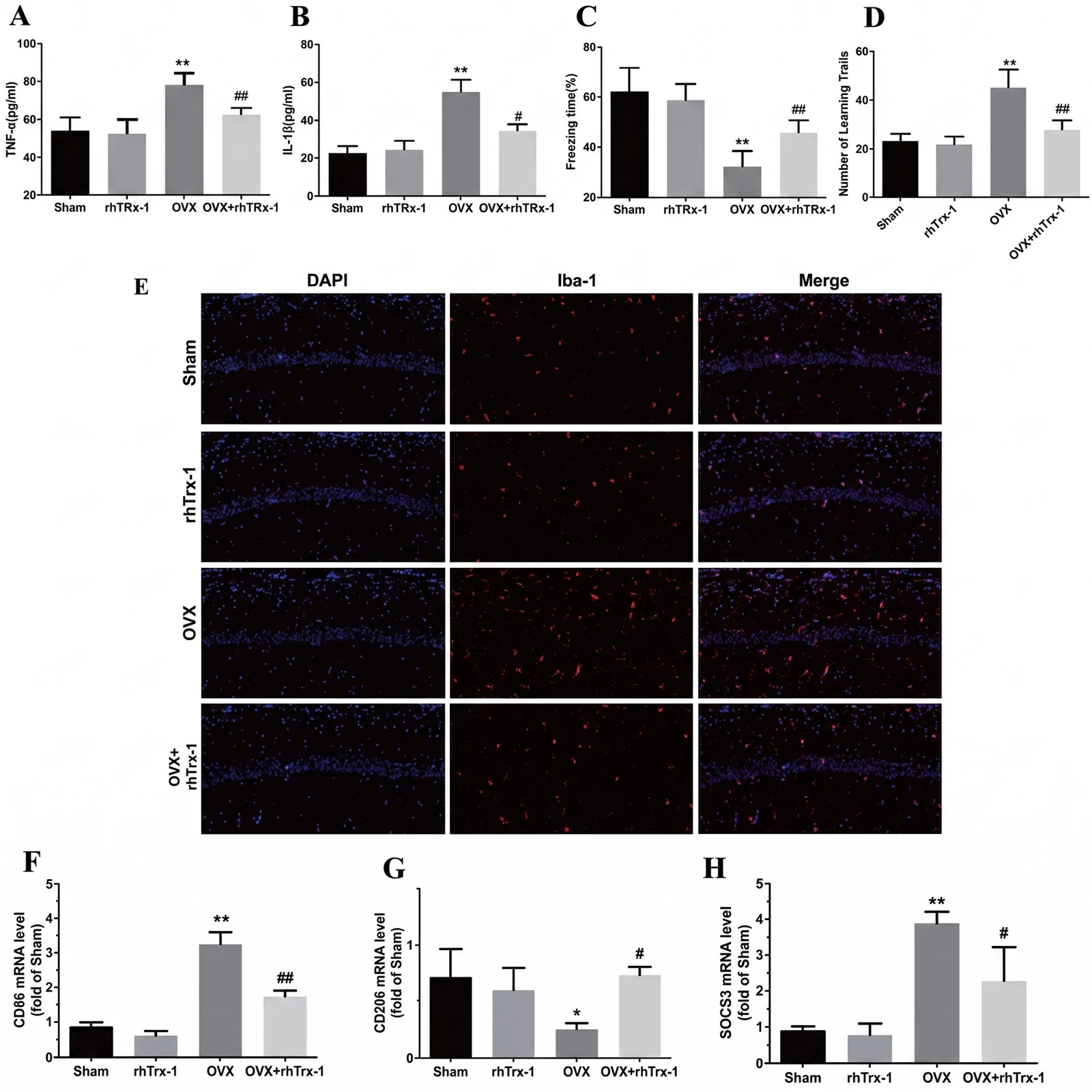
rhTrx-1 administration inhibited OVX-induced neuroinflammation and alleviated cognitive decline. (**A, B**) TNF-α and IL-1β levels were measured by ELISA. (**C**) Freezing time was recorded to assess the contextual fear response. (**D**) Y-maze test was conducted to evaluate spatial learning and memory. (**E**) Iba-1 immunofluorescence in the CA1 region of the hippocampus was used to assess microglial activation. Scale bar = 50 μm. (**F-H**) M1 (CD86), M2a (CD206), and M2b (SOCS3) phenotype markers were detected by real-time PCR. Error bars: ± SD; Statistical test: one-way ANOVA with Tukey’s post hoc test, *P < 0.05, **P < 0.01 *versus* Sham mice; ^#^*P* < 0.05, ^##^*P* < 0.01 *versus* OVX mice. Sample size: n = 10 mice per group.

To further investigate the impact of Trx-1, rhTrx-1 was administered *via* intraperitoneal injection weekly after ovariectomy in the OVX group. This treatment suppressed proinflammatory TNF-α and IL-1β secretion (Figs. **5A**, **B**) and ameliorated cognitive impairment (Figs. **5C**, **D**). Additionally, rhTrx-1 administration significantly inhibited microglial activation in the hippocampus (Fig. **5E**).

To determine whether rhTrx-1 altered microglial phenotype in the hippocampus, we examined specific microglial markers. OVX upregulated M1 (CD86) and immunomodulatory M2b (SOCS3) markers, while downregulating the M2a repair/regeneration marker (CD206), indicating microglial activation. Treatment with rhTrx-1 reversed this M1/M2 phenotypic shift (Figs. **5F**-**H**). Collectively, these findings indicate the potential therapeutic efficacy of Trx-1 in alleviating menopausal cognitive impairment.

### 3.5 Administration of rhTrx-1 Effectively Suppresses LPS-induced Microglial Activation *In vitro*

**Fig. (6):**
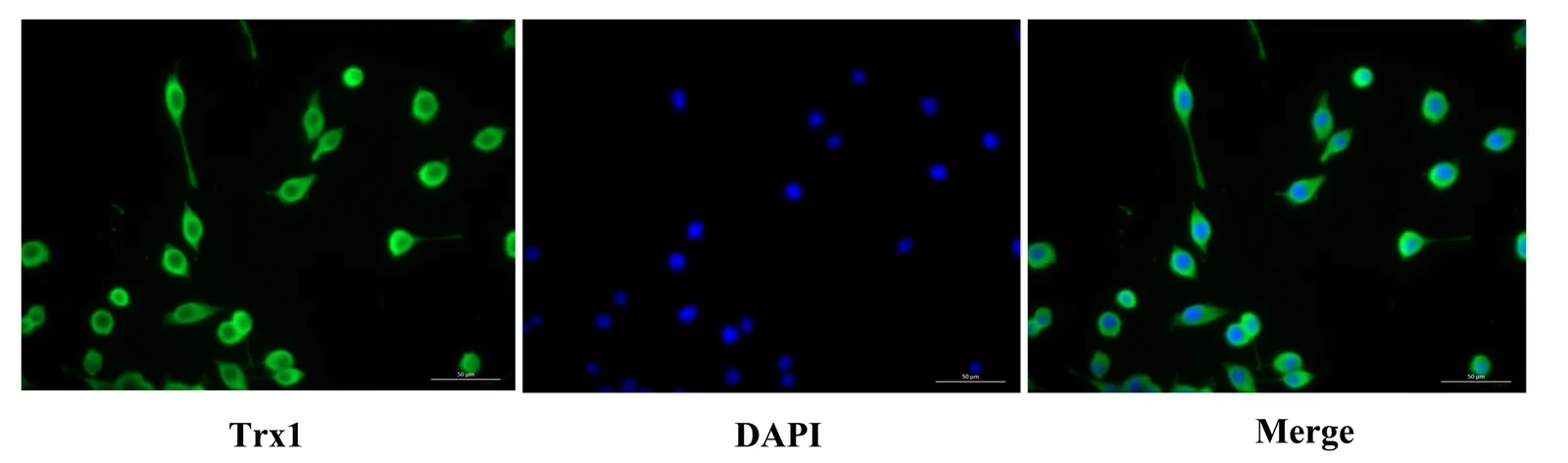
Abundance of Trx-1 in BV2 cells. IF staining of Trx-1 (green) in BV2 cells. The nuclei were stained with DAPI (blue). Scale bar = 50 μm.

Given the clear indication of a correlation between Trx-1 reduction and neuroinflammation after menopause, we conducted in vitro experiments using BV-2 cells to elucidate the protective effect of rhTrx-1 on microglial activation. Immunofluorescence staining showed the presence and location of Trx-1 in BV2 cells (Fig. **6**).

**Fig. (7):**
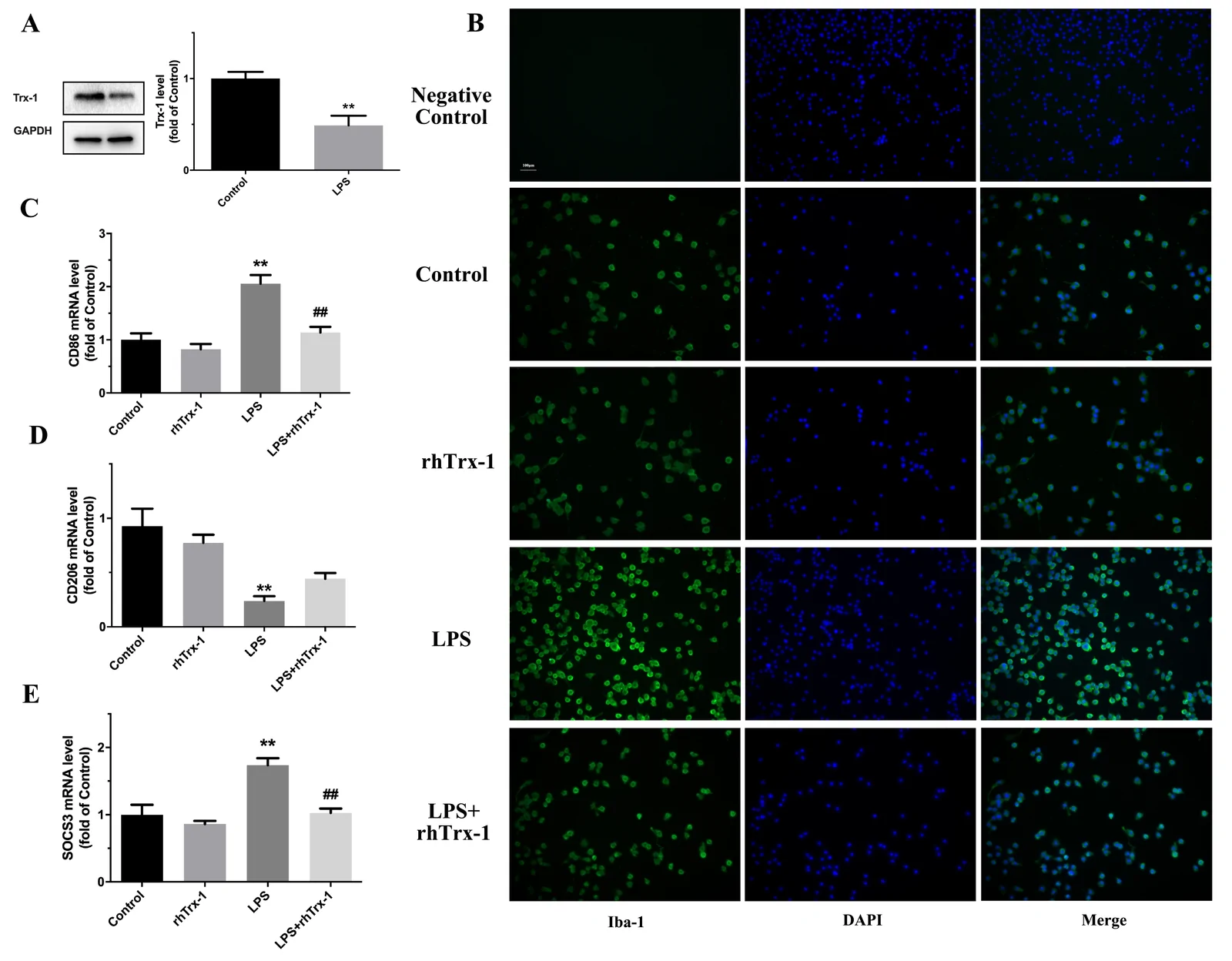
Administration of rhTrx-1 inhibited LPS-induced microglia activation. (**A**) The expression of Trx-1 of microglial cells was detected by Western blotting. (**B**) Iba-1 immunofluorescent staining (green) was used to test the activated levels of BV-2 microglial cells with confocal scanning, and DAPI staining of the nucleus (blue). Scale bar = 100 μm. (**C-E**) M1(CD86), M2a (CD206), and M2b (SOCS3) phenotype markers were detected by Real-time PCR. Error bars: ±SD; Statistical test: Student's t-test (A) or a one-way ANOVA with Turkey's post hoc test (**C-E**), **P<0.01 *versus* Control group. ^##^*P*<0.01 *versus* LPS group; Sample size: n=3 independent experiments.

As depicted in Fig. (**7A**), Trx-1 expression in BV-2 cells was diminished by LPS treatment at a concentration of 100 ng/ml. Furthermore, consistent with previous investigations, LPS significantly triggered the activation of microglia. However, pretreatment with rhTrx-1 at a concentration of 25 μg/ml for a duration of 30 minutes effectively inhibited microglial activation (Figs. **7B**-**E**).

### 3.6 Mitogen-Activated Protein Kinases (MAPK) Signaling Pathways were Implicated in the Protective Role of Trx-1

**Fig. (8):**
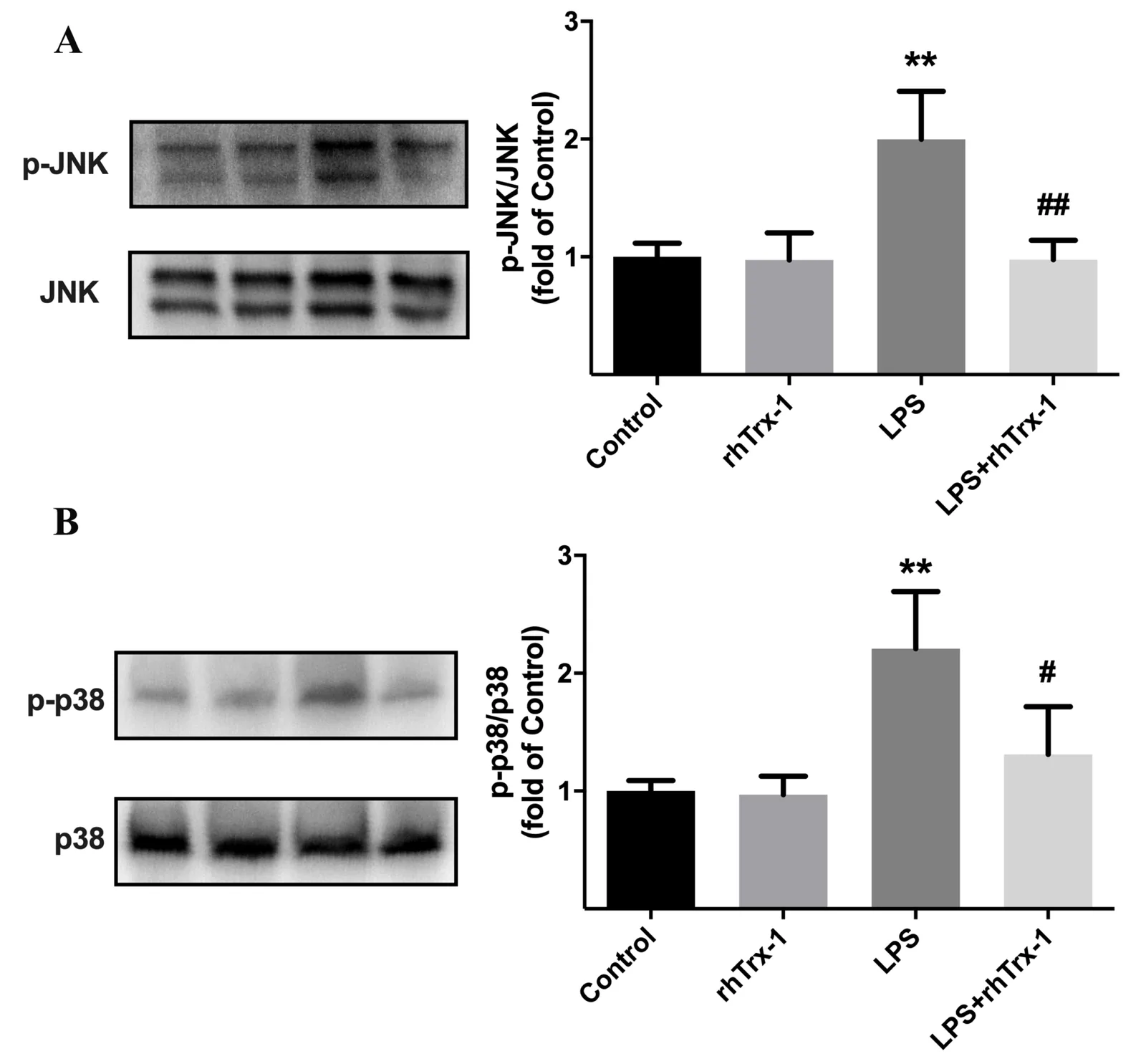
Trx-1 inhibited LPS-induced MAPK activation. The activated levels of JNK (**A**) and p38 (**B**) were assessed. Error bars: ± SD; Statistical test: a one-way ANOVA with Turkey's post hoc test, **P<0.01 *versus* the Control group. ^#^*P*<0.05 *versus* LPS group, ^##^*P*<0.01 *versus* LPS group; Sample size: n=3 independent experiments.

**Fig (9):**
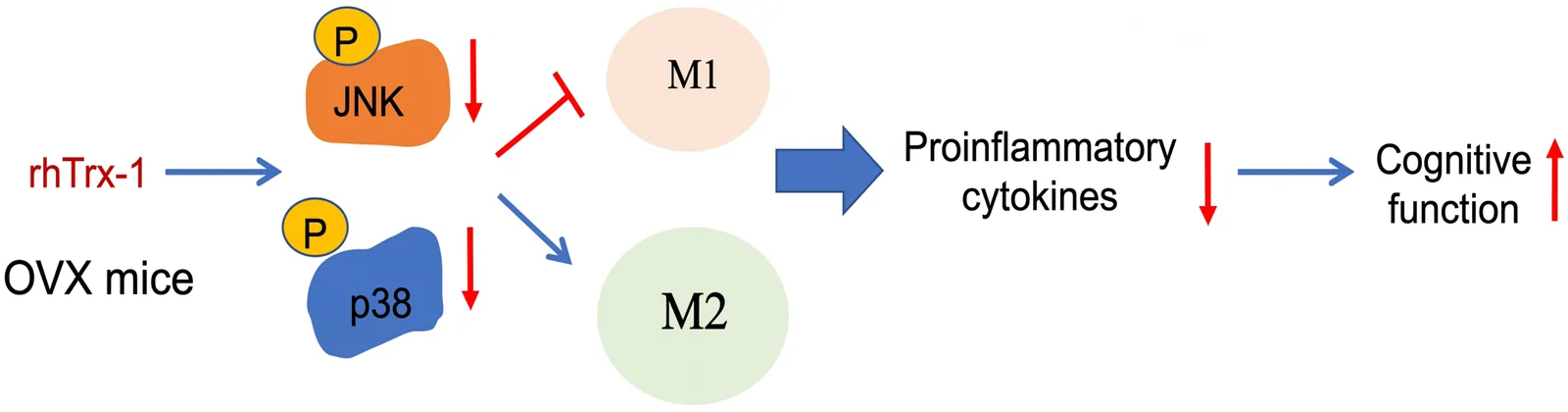
Role of Trx-1 in ameliorating cognitive impairment in OVX mice. The production of inflammatory factors and microglial activation induced by OVX contribute to cognitive deficits in mice. While Trx-1 recombinant protein drives a shift in microglial polarization toward the M2 phenotype, potentially through inhibition of the MAPK signaling pathway, thereby decreasing proinflammatory cytokine release and rescuing cognitive function in OVX mice.

As previously reported, stimulation of microglial cells with LPS (100 ng/ml) resulted in significant phosphorylation of JNK (Fig. **8A**) and p38 (Fig. **8B**). However, pretreatment with rhTrx-1 (25 μg/ml) for 30 minutes effectively reversed this phenomenon. A schematic diagram is constructed to illustrate the possible mechanism by which Trx-1 modulates cognition (Fig. **9**).

## 4 DISCUSSION

The global population aged over 60 is projected to reach 2 billion by 2050 [29], and this demographic shift is accompanied by an increased prevalence of cognitive decline. Postmenopausal senile dementia represents one of the most significant forms of dementia. Epidemiological studies indicate that postmenopausal females have a higher incidence of dementia than males of the same age, suggesting a sex difference in neurological disorders [30, 31]. Numerous studies have confirmed that women who undergo surgical menopause at an early age experience accelerated cognitive decline and increased Alzheimer's disease (AD) neuropathology [5-7].

Surgical menopause is a fundamental approach for managing premenopausal hormone receptor-positive breast cancer and is essential both for treating ovarian malignancies and for reducing cancer risk in susceptible populations [1, 2]. With improving survival outcomes for malignancies such as breast and ovarian cancer, preserving long-term quality of life for survivors has become increasingly important. Although estrogen has potential neuroprotective effects, some clinical trials suggest that menopausal hormone therapy may not improve—and could even impair—cognitive function in postmenopausal women. A randomized clinical trial found no significant cognitive benefits of estrogen therapy in recently postmenopausal women [12]. In addition, a case-control study involving 84,739 postmenopausal women reported that those with AD were more likely to have used systemic estradiol [11], with elevated risk specifically linked to hormone therapy exposure exceeding 10 years [11]. Long-term estrogen therapy has also been associated with increased risks of venous thromboembolism, stroke, breast cancer, and ovarian cancer [32]. Therefore, identifying novel therapeutic targets for cognitive impairment in postmenopausal women is crucial.

A surgical menopause animal model was established by removing the ovaries of mice [22]. Our findings align with those of Aggarwal *et al*., who reported that mice exhibited low estradiol levels and cognitive deficits four weeks after ovariectomy [23]. Our experimental model successfully reproduces this medically induced menopausal condition. The present study demonstrates that ovarian removal, reflecting therapeutic oophorectomy in humans, initiates a sequential process of hippocampal inflammation. This neuroinflammatory response, characterized by microglial activation and elevated pro-inflammatory signaling, ultimately results in measurable impairments in learning and memory. These observations provide a credible biological foundation for cancer therapy-related cognitive impairment reported by patients after ovarian suppression [4].

While our study demonstrated the neuroprotective role of Trx-1 in estrogen deficiency-induced cognitive dysfunction, we did not experimentally examine this association in microglia. The link between estrogen and Trx-1 is supported by several existing studies in various cell types [25, 33, 34]. In human umbilical vein endothelial cells, intermittent hypoxia downregulated both mRNA and protein expression of Trx-1, whereas 17β-estradiol (E2) promoted its expression [33]. Similarly, treatment with 17β-estradiol at concentrations of 10 nM and 10 μM increased Trx-1 protein levels in retinal ganglion cells [25]. An independent study further confirmed the consistent upregulation of both Trx-1 protein and its corresponding mRNA following 17β-estradiol treatment in primary stromal cells derived from human endometrium [34].

Although the molecular mechanism is not fully elucidated, it is known to involve estrogen receptor signaling. Notably, the canonical estrogen-responsive element (ERE) is absent in the Trx promoter [35-37], suggesting that E2-induced Trx gene activation likely occurs downstream of primary transcriptional effects. However, previous reports indicate that imperfect ERE half-sites, specificity protein 1 (Sp1), and activator protein-1 (AP-1) sites within the 5'-flanking region of the Trx gene may function as alternative EREs to mediate transcriptional activation upon E2 stimulation [37-39].

To further elucidate the precise mechanism by which estrogen regulates Trx-1 expression, future studies are warranted. We plan to employ BV-2 microglial cells to determine the dose- and time-dependent effects of E2 on Trx-1 levels and to verify estrogen receptor (ER) dependency using specific antagonists. Subsequently, the Trx promoter will be cloned into a luciferase reporter vector to directly test its responsiveness to E2 and to map critical regulatory elements, such as AP-1 and Sp1 sites, through deletion and site-directed mutagenesis analyses. Finally, chromatin immunoprecipitation (ChIP) assays will be utilized to identify the specific transcription factors recruited to the Trx promoter upon E2 stimulation.

 Microglia are innate immune cells capable of triggering inflammatory responses through the excessive release of IL-1β and TNF-α. Microglial activation has been linked to numerous neurodegenerative diseases [40]. We observed that ovariectomy resulted in upregulation of CD86 and IL-1β, markers associated with the cytotoxic M1 phenotype, as well as SOCS3, a marker indicative of the immunomodulatory M2b phenotype. Conversely, CD206, a marker related to the M2a phenotype involved in tissue repair and regeneration, was downregulated in OVX mice. These findings are consistent with a recent investigation demonstrating that OVX induces a shift in microglial polarization from an immunoregulatory M2 phenotype to a proinflammatory M1 phenotype [24]. Therefore, modulating microglial M1/M2 phenotypes may represent a promising therapeutic strategy. Our study demonstrates that Trx-1 administration exerts neuroprotective effects against cognitive impairment in OVX mice by attenuating microglial activation.

Exogenous administration of recombinant human Trx-1 exhibits anti-inflammatory effects *in vivo*. Trx-1 not only protects cells from oxidative and inflammatory stress but also plays a crucial role in gene transcription and signal transduction [41]. Decreased Trx-1 levels have been implicated in neurodegenerative mechanisms underlying AD [42]. Overexpression of the Trx-1 gene in transgenic mice improved spatial learning and memory deficits associated with diabetic encephalopathy by inhibiting proteins involved in the protein kinase RNA-like ER kinase (PERK) and inositol-requiring enzyme 1α (IRE1α) pathways [43]. Ectopic expression of Trx-1 markedly enhanced proliferation of non-small cell lung cancer epithelial cells while attenuating hydrogen peroxide (H_2_O_2_)-induced oxidative damage. Moreover, Trx-1 overexpression effectively suppressed rapid activation of JNK and p38 MAPK signaling pathways [44]. Our previous study observed that LPS stimulation of microglia led to increased JNK phosphorylation [27]. The study also found decreased Trx-1 levels in the hippocampus of OVX mice compared to sham mice. These findings suggest that Trx-1 may be involved in menopause-related dementia through the MAPK pathway. Consistent with this hypothesis, we discovered that Trx-1 mitigated microglial activation and polarization by inhibiting phosphorylation of JNK and p38.

Thyroid hormones play a pivotal role in human development, critically regulating fetal and neonatal brain maturation, driving somatic growth throughout childhood and adolescence, and maintaining metabolic homeostasis later in life [45]. The ontogeny of fetal thyroid function follows a precise timeline, with maternal free thyroxine (FT4) serving as the sole source until the maternal-to-fetal transition occurs around mid-gestation [46, 47]. Accumulating evidence indicates that perturbations in maternal thyroid hormone concentrations during early gestation can exert long-term effects on offspring neurodevelopment [48, 49]. Maternal FT4 levels in early pregnancy have been identified as significant predictors of axonal microstructure in the white matter of male offspring in early adulthood, suggesting a role of early gestational thyroid status in the developmental programming of long-term brain connectivity [48]. Another study in children reported that maternal hypothyroidism during pregnancy was associated with smaller hippocampal volume in offspring, a morphological difference that correlated with memory performance [49].

Nuclear magnetic resonance-based metabolomic analysis of 90 matched maternal serum and colostrum samples revealed that hypothyroidism significantly alters metabolite profiles during pregnancy and lactation. These metabolic perturbations point to potential disruptions in lipid and glucose pathways, which may contribute to aberrant neurodevelopment and predispose offspring to later-life metabolic syndrome or neurodegenerative disorders [50]. Thus, hypothyroidism during pregnancy represents a prevalent endocrine dysfunction that directly perturbs metabolic homeostasis, establishing it as a significant mediator of cognitive impairment.

The present study demonstrates that estrogen deficiency is a key driver of neuroinflammation and cognitive decline. Estrogen deficiency has been shown to perturb the hypothalamic-pituitary-thyroid axis and alter thyroid hormone metabolism [51]. Critically, both estrogen and thyroid hormones are pivotal regulators of cognitive processes. We propose that this decline may interact with other hormonal systems, including thyroid function and cellular redox networks (*e.g*., Trx-1), thereby creating a vulnerable milieu for neuroinflammation and cognitive impairment.

## 5 STUDY LIMITATIONS

Several limitations should be considered in our study. Although our data demonstrate decreased Trx-1 levels in OVX mice, the molecular mechanism underlying E2-mediated regulation of Trx-1 was not investigated. A previous study revealed that 17β-E2 provided protection against iron-induced injury in the female brain, while this neuroprotective effect was attenuated by Trx-1 small interfering RNA (siRNA) [52]. To establish the *in vivo* relevance of E2 and Trx-1, future studies will co-administer E2 with an ER antagonist in OVX mice and assess the effects on both Trx-1 expression and cognitive function. To delineate the specific ER subtypes and transcription factors involved, OVX mice will be treated with ERα- or ERβ-selective agonists, followed by administration of specific transcription factor inhibitors to evaluate Trx-1 expression and its protective effects.

To obtain direct evidence of transcriptional regulation, ChIP assays will be performed on hippocampal tissues to investigate the estrogen-dependent recruitment of ER and specific transcription factors to the Trx promoter. Furthermore, studies using Trx-1 transgenic mice could provide a broader understanding of the role of Trx-1 in neuroinflammation under OVX conditions. While OVX mice represent a classic menopausal model, they may not fully generalize to other species or all clinical conditions. Therefore, additional animal models are required to validate our results.

## CONCLUSION

The current investigation demonstrates that OVX surgery induces microglial activation and proinflammatory cytokine secretion, resulting in neuroinflammation and cognitive impairment. Administration of rhTrx-1 inhibited microglial activation *via* MAPK pathways, thereby alleviating cognitive deficits in OVX mice. These findings suggest that Trx-1 may represent a potential therapeutic strategy for postmenopausal cognitive dysfunction. Nevertheless, future studies employing additional animal models and Trx-1 transgenic approaches will be essential to validate these results.

## Data Availability

The analyzed data sets generated during the study are available from the corresponding author upon reasonable request.

## LIST OF ABBREVIATIONS

HR: Hormone Receptor
BRCA1/2: Breast Cancer Susceptibility Gene 1/2
ELISA: Enzyme-Linked Immunosorbent Assay
OVX: Ovariectomy
PCR: Polymerase Chain Reaction
rhTrx-1: Recombinant Human Trx-1
LPS: Lipopolysaccharide
MAPK: Mitogen-activated Protein Kinases
Trx: Thioredoxin
CO_2_: Carbon Dioxide
PBS: Phosphate Buffered Saline
TFC: Trace Fear Conditioning
FBS: Fetal Bovine Serum
DMEM: Dulbecco's Modified Eagle's Medium
Iba1: Ionized Calcium-binding Adapter Molecule 1
DAPI: 4',6-diamidino-2-phenylindole
RT-qPCR: Reverse-transcription Quantitative Real-time Polymerase Chain Reaction
CD86: Cluster of Differentiation 86
CD206: Cluster of Differentiation 206
SOCS3: Suppressor of Cytokine Signaling 3
BSA: Bovine Serum Albumin
JNK: c-Jun N-terminal Kinase
TNF-α: Tumor Necrosis Factor α
IL-1β: Interleukin-1β
SD: Standard Deviation
CA1: Cornu Ammonis 1
AD: Alzheimer's Disease
PERK: Protein Kinase RNA-like ER Kinase
IRE1α: Inositol-requiring Enzyme 1α
H_2_O_2_: Hydrogen Peroxide
17β-E2: 17β-estradiol
siRNA: Small Interfering RNA
ERE: Estrogen-responsive Element
Sp1: Specificity Protein 1
AP-1: Activator Protein-1
ER: Estrogen Receptor
ChIP: Chromatin Immunoprecipitation
FT4: Free Thyroxine
